# Editorial: Surface enhanced raman scattering: Theory and applications

**DOI:** 10.3389/fchem.2023.1176495

**Published:** 2023-04-27

**Authors:** Joydeep Chowdhury, Václav Ranc, Sougata Sarkar

**Affiliations:** ^1^ Jadavpur University, Kolkata, India; ^2^ Palacký University, Olomouc, Czechia; ^3^ Ramakrishna Mission Vivekananda Centenary College, Rahara, India

**Keywords:** SERS, plasmons, sensors, DFT, nanoparticles

Surface-enhanced Raman scattering (SERS) has turned out to be a fascinating analytical tool that can detect analyte molecules at trace concentrations. The technique though established as a formidable analytical tool continued to draw research interests among the physicists, chemists, material scientists and biologists. The primary reason behind the fact lies in understanding the phenomenon and fabrications of durable SERS responsive substrates which in turn stand responsible for the generation of SERS sensors towards the detection of explosives, illicit drugs and in diagnostics. We are deeply privileged to compile this Research Topic of *Frontiers in Chemistry* dedicated to “*Surface Enhanced Raman Scattering: Theory and Applications* **”** that vividly depict all the modern aspects in this area of research both from the viewpoint of fundamental aspects to its applications. The seven invited articles in this Research Topic cover the recent advances in SERS research covering all its aspects in the current scenario.

The first article by Zhang et al. is dedicated to the fabrication details of SERS active substrate with silver nanoparticles (AgNPs) decorated on colloidal polystyrene (PS) microspheres through a seed-mediated *in situ* growth technique. This technique helps to generate highly dense agglomeration of AgNPs on the surface of the colloid that yields superior SERS enhancement. They showed that the PS/Ag substrate can sense Nile blue A (NBA) at a low concentration ∼10^–7^ M. Detection of malachite green (MG) from fish was also accomplished with the limit of detection (LOD) 0.02 ppm. Furthermore the substrate also shows its ability to sense many pesticides simultaneously by SERS technique.

The second article by Das et al. is focused on Raman, SERS and surface enhanced resonance Raman scattering (SERRS) studies of merocyanine dye supported by DFT calculations. Existences of the two different conformeric forms of the molecule have been suggested. The adsorptive behaviour of the dye on as prepared silver-coated films (SCFs) were investigated from wavelength dependent SERS studies.

The third article by BrezesteanFarcău et al. is focused on the fabrication of colloidal silver nanoparticle (AgNP) films by convective self-assembly technique. The nanoparticles so fabricated were subsequently used for a detection of pesticide α-endosulfan (α- ES). SERS efficacy of the AgNPs films is tested with p-aminothiophenol molecules. They also showed that the pesticide Thiabendazole could be readily adsorbed on the prepared AgNPs films. The AgNPs films can detect the presence of Thiabendazole at a low concentration down to 10^–6^ M. Efficacies of the substrates for detecting the pesticides were further demonstrated from multivariate data analyses.

The fourth article by Zhang et al. discusses about SERRS and fluorescent sensor for detection of a plant harmone Abscisic acid (ABA). The dual SERRS and fluorescence sensor was developed based on the quenching of Raman enhancement and fluorescence quenching properties of gold nanorods (AuNRs).

The fifth article by Zhai et al. is devoted on the fabrication of arrays of high density silver nanoparticles-decorated TiO_2_ nanotubes as three dimensional reusable SERS active substrate for the detection of organic dye molecules. The enhancement factor (EF) of the as prepared SERS substrate was reported to be as ∼ 1.4×10^8^ orders of magnitude, thus showing promising potentials for rapid and trace SERS detections of organic chemicals.

The sixth article by Sinha et al. is aimed on the fabrication of SERS active substrate through self-assembly of Gold nanoparticles on Heat cooled Calf Thymus DNA (HC-Ct DNA) Langmuir-Blodgett (LB) film scaffold. Substrate shows huge enhancement of Raman bands of 4-mercaptopyridine molecule upon adsorption of the gold nanoparticles embedded DNA origamic scaffolds. The substrate has also been used for sensing pesticide malathion at trace concentration.

Final article written by Si et al. discusses about the adsorption of the 5-fluorouracil (5FU) on small gold clusters AuN by means of SERS and DFT. Theoretical calculations show that N-H and C=O stretching vibrations play a pivotal role in the SERS phenomenon. Mechanism for the releasing drug from the gold surface is also depicted.

This Research Topic contains the original studies covering all modern aspects of research in the field of surface enhanced Raman spectroscopy. We are deeply indebted to all the authors for their wonderful contributions and sharing their recent researches in this Research Topic. Special thanks to Dr. Subhendu Chandra, Associate Professor in Physics, Victoria Institution (College), Kolkata, India and for his kind help, suggestions and secretarial assistance throughout the editing process.

We are deeply grateful to all the learned reviewers for critically reviewing the manuscripts in time despite of their busy schedule. Finally we will feel that our venture to be fruitful if the contents of this Research Topic prove useful and thought provoking not only for the experts but also for the young researchers who are new in this exciting field of research.

